# Effects of Stingless Bee Propolis on Experimental Asthma

**DOI:** 10.1155/2014/951478

**Published:** 2014-04-01

**Authors:** José Hidelbland Cavalcante de Farias, Aramys Silva Reis, Marcio Antonio Rodrigues Araújo, Maria José Abigail Mendes Araújo, Anne Karine Martins Assunção, Jardel Cavalcante de Farias, Eder Magalhães Silva Fialho, Lucilene Amorim Silva, Graciomar Conceição Costa, Rosane Nassar Meireles Guerra, Maria Nilce Sousa Ribeiro, Flávia Raquel Fernandes do Nascimento

**Affiliations:** ^1^Laboratory of Immunophysiology, Department of Pathology, Biological and Health Sciences Center, Federal University of Maranhão (UFMA), 65085-580 São Luís, MA, Brazil; ^2^Laboratory of Pharmacognosy, Department of Pharmacy, Biological and Health Sciences Center, Federal University of Maranhão (UFMA), 65085-580 São Luís, MA, Brazil

## Abstract

Bee products have been used empirically for centuries, especially for the treatment of respiratory diseases. The present study evaluated the effect of treatment with a propolis hydroalcoholic extract (PHE) produced by* Scaptotrigona * aff. *postica* stingless bee in a murine asthma model. BALB/c mice were immunized twice with ovalbumin (OVA) subcutaneously. After 14 days, they were intranasally challenged with OVA. Groups P50 and P200 received PHE by gavage at doses of 50 and 200 mg/kg, respectively. The DEXA group was treated with intraperitoneal injection of dexamethasone. The OVA group received only water. The mice were treated daily for two weeks and then they were immunized a second time with intranasal OVA. The treatment with PHE decreased the cell number in the bronchoalveolar fluid (BAL). Histological analysis showed reduced peribronchovascular inflammation after treatment with PHE especially the infiltration of polymorphonuclear cells. In addition, the concentration of interferon-**γ** (IFN-**γ**) in the serum was decreased. These results were similar to those obtained with dexamethasone. Treatment with* S.* aff* postica* propolis reduced the pathology associated with murine asthma due an inhibition of inflammatory cells migration to the alveolar space and the systemic progression of the allergic inflammation.

## 1. Introduction

Asthma is considered a major public health problem that affects approximately 10% of the world's population. It is a chronic inflammatory disease of the airways in which many cells play an important role [1]. Airway wall infiltration by mast cells, eosinophils, dendritic cells, macrophages, neutrophils, and T lymphocytes can be observed in lung inflammatory responses, which are associated with the increased expression of multiple proteins involved in a complex inflammatory cascade mediated by cytokines, chemokines, and lipid mediators [2].

This disease may present an immediate hypersensitivity reaction followed by a late response phase. In experimental models, the immediate phase can be adoptively transferred by serum, whereas the late phase is transferred by CD4^+^ T helper type 2 (Th2) lymphocytes. Both IgE and mast cells are crucial for triggering the immediate allergic phase. In the late phase, the involvement of Th2 cells is critical as they are responsible for the release of cytokines, including interleukins (IL) IL-4, IL-5, IL-9, and IL-13, which, in turn, are responsible for the eosinophilic inflammation [3]. Despite the knowledge about the role of Th2 cytokines and IgE in the experimental models of asthma, it has been shown that the Th1, associated with Th2 response, is also important in the pulmonary damage. The Th1 profile* per se* does not induce any characteristic of asthma, but the interferon-*γ* (IFN-*γ*) has been associated with pathogenesis of asthma and the severity of this disease [4]. In an asthma model, it was shown that the administration of neutralizing antibody to IFN-*γ* suppresses the airway hyperactivity, being justifiable inhibition of this cytokine for the treatment of asthma [5].

Currently, the glucocorticoids are the most effective treatment for asthma and have been proven to be safe. Their efficacy has been well documented in preventing morbidity and mortality associated with asthma and in improving disease prognosis, including reducing hospitalisations, preventing relapse, and promoting recovery, especially in patients with severe asthma and in children [6]. However, their prolonged systemic use can cause many undesirable side effects associated with the products of their catabolism, an immunosuppression [7]. There is still the ambitious goal in the pharmaceutical industry to produce steroidal analogs that avoid the side effects and maintain the therapeutic efficacy [8].

So murine experimental asthma models have been developed using various types of known allergens [9, 10] and have greatly contributed to study the inflammatory mechanisms of asthma and to test new drugs, especially those derived from the empirical use of natural products such as those produced by bees, in attempts to minimise the discomfort that occurs during asthma attacks.

Propolis produced by Africanized honeybees is a potent anti-inflammatory agent in acute and chronic inflammation, which has been confirmed through* in vitro* and* in vivo* experiments with ethanolic and aqueous propolis extracts or with compounds isolated from propolis [11–14].

It has been shown that components derived from propolis activate macrophages and that the polyphenols may be responsible for increasing macrophage capacity to phagocyte to stimulate lymphocytes and to kill microorganisms and tumours [14–16]. An important fact is that despite activating macrophages and inducing the release of free radicals, polyphenols are widely known as antioxidants [17, 18] that sequester excess free radicals generated by macrophages and neutrophils [19].

Although much is already known about the therapeutic action of propolis produced by Africanized honeybees, there has been little investigation about the therapeutic properties of propolis produced by stingless bees. Therefore, the present work evaluated the effect of propolis produced by* Scaptotrigona* aff.* postica,* a stingless bee, on pulmonary inflammation due to an experimental asthma induced in mice.

## 2. Material and Methods

### 2.1. Preparation of Propolis Hydroalcoholic Extract (PHE)

Propolis produced by* Scaptotrigona* aff.* postica* was collected from the internal parts of a beehive located in the municipality of Barra do Corda (05°30′20′′S, 45°14′36′′W), state of Maranhão, Brazil. The in natura propolis was extracted by maceration in ethanol (70%) for 24 h. The extractive solution was filtered and concentrated to a small volume at 40°C in a rotary evaporator under low pressure, obtaining the hydroalcoholic extract of propolis (PHE). The dry weight was calculated yielding 9 g of product (4%) [16]. Flavonoids, phenolic acid, and total phenol contents found in the PHE were 0.55 ± 0.07%, 11.40 ± 0.73%, and 11.95 ± 0.80%, respectively [20].

### 2.2. Animals

Adult female BALB/c mice aged from two to three months (*n* = 5/group) were provided by the Animal Facility of the Federal University of Maranhão. During the studies, the animals were maintained in the Animal Facility of the Immunophysiology Laboratory under controlled environmental conditions. Both water and food were offered* ad libitum* until the day of sacrifice. The animals were handled in compliance with the ethical norms established by the Brazilian College of Animal Experimentation and the present project was approved by the Ethics Committee on Animal Research of the Maranhão State University (Protocol number 010/2007).

### 2.3. Ovalbumin- (OVA-) Induced Allergic Pulmonary Inflammation

The animals were immunized subcutaneously (sc.) with 4 *μ*g OVA adsorbed onto 1.6 mg aluminium hydroxide (alum). After seven days, the same procedure was repeated. After another seven days, the animals were lightly anesthetised with 0.4 mL of a xylazine hydrochloride (20 mg/kg) solution and were challenged by intranasal instillation (in.) with a 50 *μ*L OVA solution (10 *μ*g OVA/50 *μ*L sterile phosphate-buffered saline (PBS)). Seven days after the first challenge, the animals were challenged again with the same solution [21]. After 24 hours, blood was collected to obtain the serum, and the animals were euthanized using lethal i.p. injection of 10% chloral hydrate.

### 2.4. Treatment of Allergic Pulmonary Inflammation with PHE

Treatments were initiated immediately after the second immunisation. The animals were treated orally with 100 *μ*L PHE at doses of 50 or 200 mg/kg/animal (P50 and P200, resp.) for 14 consecutive days. Animals in the positive control group received i.p. injections of 100 *μ*L dexamethasone (DEXA) at 1 mg/kg/animal for 14 consecutive days. The negative control group (OVA) received only orally administered saline solutions. The clean control was neither sensitised nor challenged.

### 2.5. Collection and Counting of Bronchoalveolar Lavage (BAL) Cells

For each animal, the animal's trachea was exposed and 0.5 mL of cold PBS was injected in the bronchoalveolar space. After BAL aspiration, another 0.5 mL PBS was injected and aspirated. To determine the total BAL cell number, 90 *μ*L of the cell suspensions was fixed and stained in 10 *μ*L solution containing 0.05% crystal violet diluted in 30% acetic acid. Subsequently, the cells were counted in a Neubauer chamber with the aid of an optical microscope at 400x magnification.

### 2.6. Lung Histopathological Evaluation

After BAL collection, the lungs were perfused with 10 mL PBS through a cannula inserted into the right ventricle to remove residual blood, and the lungs were weighed and fixed by immersion in buffered formalin (10%). After 24 hours, the organs were transferred to a 70% alcohol solution until paraffin embedding. The tissues were cut into 5 *μ*m sections and stained with haematoxylin/eosin for histopathologic examination. The inflammatory process in the histological sections was qualitatively evaluated and characterised as absent, mild, moderate, or severe according to the characteristics of the affected area.

### 2.7. Collection and Counting of Cells from the Peritoneal Lavage and Lymphoid Organs

To verify if the effects of PHE observed in lung could be due a systemic immunosuppression, we evaluated lymphoid organs and also the peritoneal cavity. The animal's peritoneal cavity was washed with 5 mL sterile PBS. After abdominal wall excision, cell suspensions were obtained by aspiration using a syringe and needle, transferred to conical-bottom polypropylene tubes, and maintained in an ice bath (4°C) until the cells were counted. After collection of the peritoneal lavage, the spleen and mesenteric lymph nodes were collected, weighed, and crushed. The femur was perfused with 1 mL PBS to obtain bone marrow cells.

For total cell number counting, 90 *μ*L of each cell suspension was fixed and stained with 10 *μ*L 0.05% crystal violet in 30% acetic acid. The cells were counted using a Neubauer chamber with the aid of an optical microscope at 400x magnification.

### 2.8. Quantification of Serum Interferon Gamma (IFN-*γ*)

IFN-*γ* has been shown to be involved in the pathogenesis of asthma and can be found in atopic patients [4]. So we quantified this crucial cytokine in the serum. The quantitation was performed in 96-well flat-bottom microliter plates (Costar) that were coated by addition of 100 *μ*L primary antibody anti-IFN-*γ* and incubation overnight at 4°C. After incubation, the plate was inverted and washed three times with PBS+Tween 20 (PBS+T20, 300 *μ*L/well) and blocked with 200 *μ*L/well 10% foetal bovine serum (FBS) in PBS for one hour at room temperature. The wells were aspirated and washed three times with PBS+T20. Animal sera were added (100 *μ*L sample/well), and the samples were incubated for two hours at room temperature. The wells were aspirated and washed five times with PBS+T20 (300 *μ*L/well), and then 100 *μ*L/well of avidin/peroxidase-conjugated detection antibody was added. The plates were then incubated for one hour and washed seven times with PBS+T20 and 100 *μ*L/well tetramethylbenzidine substrate solution (TMB) was added. The plates were incubated for 30 minutes in the dark, and then the reaction was stopped by addition of 50 *μ*L 2 N H_2_SO_4_. Optical density reading was conducted using an enzyme-linked immunosorbant assay (ELISA) automatic reader at 450 nm absorbance (Dynatech).

### 2.9. Statistical Analysis

The analysis of the results was made using the Graph-Pad statistical software, version 5.0. Significant differences between treatments were determined by analysis of variance (ANOVA), followed by Tukey-Kramer test. Statistical significances were accepted when *P* ≤ 0.05. Data were expressed as mean ± standard deviation.

## 3. Results

### 3.1. Effect of PHE Treatment on the Number of Cells Present in the BAL of Mice Immunised and Challenged with OVA

**Figure 1:**
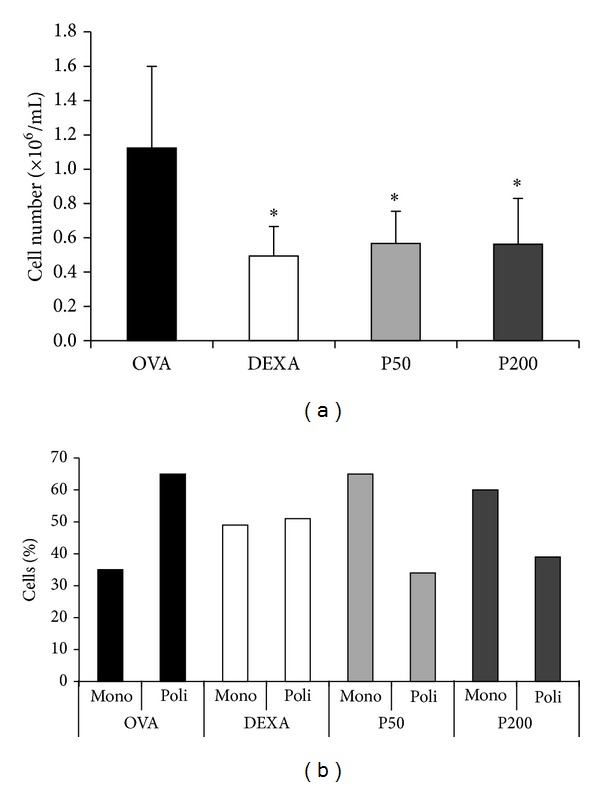
Effect of treatment with* Scaptotrigona* aff.* postica* PHE on the number of cells present in the BAL of mice immunised and challenged with OVA. Mice in groups P50 and P200 were treated orally with PHE (50 or 200 mg/kg/animal) for 14 consecutive days. The DEXA group was treated i.p. with DEXA (1 mg/kg/animal) for 14 consecutive days. The OVA group received only oral saline solution. Total (a) and differential (b) cell counts of BAL were performed 24 hours after the last challenge. *P < 0.05 compared with the OVA group.

Figure 1 shows the total and differential cell number in the BAL. There was a significant decrease in the total BAL cell number in both groups treated with PHE (P50 and P200) when compared with the OVA control group. This decrease was similar to that observed in the DEXA group (50%). There was no difference between groups DEXA, P50, and P200 (Figure 1(a)). The differential count of BAL cells demonstrated that the OVA control group showed a higher percentage of polymorphonuclear inflammatory cells (Figure 1(b)). This result was the opposite in the P50 and P200 groups, with the predominance of mononuclear cells and a percentage decrease of polymorphonuclear cells even greater than that observed in the DEXA group.

### 3.2. Effect of PHE Treatment on the Lung Histology of Mice Immunised and Challenged with OVA

**Figure 2:**
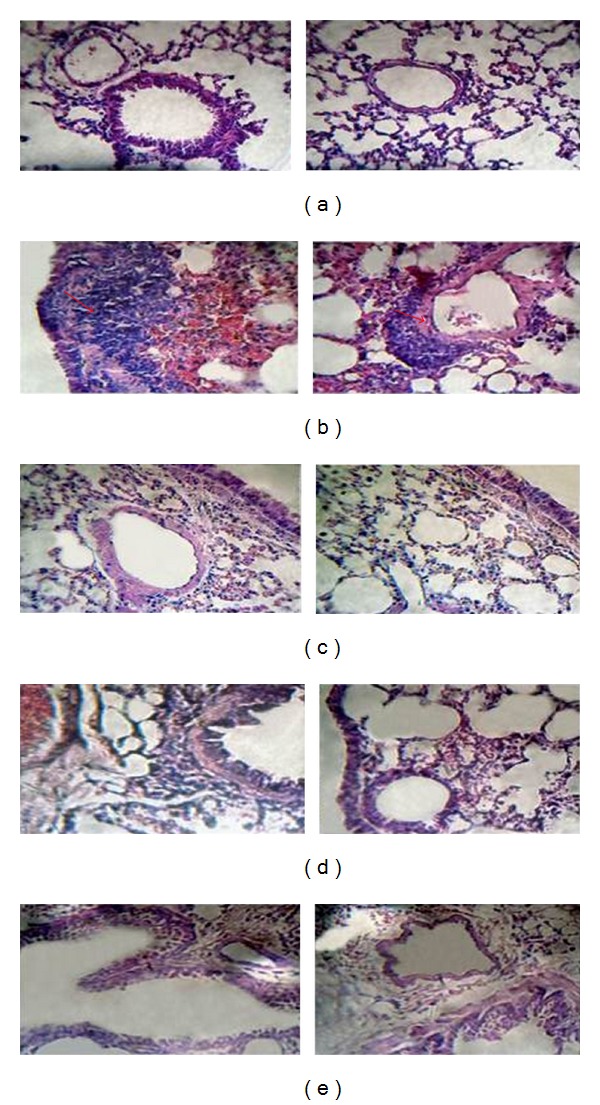
Lung histopathological sections of BALB/c mice stained with haematoxylin/eosin. Mice were immunised on days 0 and 7 with sc. injections of 4 *μ*g OVA/1.6 mg alum. On days 14 and 21, the mice were challenged by in. with 10 *μ*g OVA. The experiments were conducted 24 hours after the last challenge. Two magnifications were given (400 and 100x) to the clean control (a), OVA-immunised (b), DEXA-treated (c), and PHE-treated groups at 50 mg/kg (d) or 200 mg/kg (e). Arrows indicate areas of inflammation.

Figure 2 shows lung histological sections of mice from the different groups in photomicrographs taken at 40x magnification.  Figure 2(a) shows a section from a clean control animal, non-OVA challenged, in which a clean parenchyma without infiltration can be observed. In contrast, analysis of lung histological sections of mice in the OVA group shows intense peribronchovascular infiltration and epithelial desquamation (Figure 2(b)). DEXA (Figure 2(c)), P50 (Figure 2(d)), and P200 (Figure 2(e)) treatments restored the normal lung architecture pattern, preventing inflammatory infiltration and epithelial desquamation.

### 3.3. Effect of PHE Treatment on Circulating IFN-*γ* in Mice Immunised and Challenged with OVA

**Figure 3:**
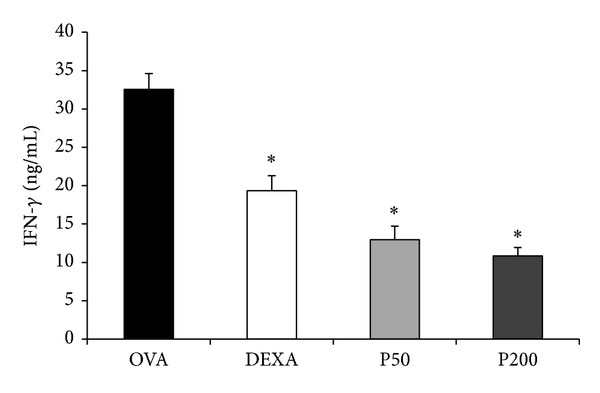
Effect of* S.* aff* postica* PHE treatment on circulating serum IFN-*γ* of mice immunised and challenged with OVA. The mice in groups P50 and P200 were treated orally with PHE (50 or 200 mg/kg/animal) for 14 consecutive days. The DEXA group was treated i.p. with DEXA (1 mg/kg/animal) for 14 consecutive days. The OVA group received only oral saline solution. The experiments were performed 24 hours after the last challenge.  *P ≤ 0.05 compared to the OVA group.

Considering the role of IFN-g in the pathogenesis of asthma, we measure this cytokine in the serum.  Figure 3 shows that the PHE-treated mice had a significant, dose-dependent decrease in serum IFN-*γ* that was more intense than that observed in the DEXA group.

### 3.4. Effect of PHE Treatment on Cell Influx into the Peritoneal Cavity and Lymphoid Organs of Mice Immunised and Challenged with OVA

**Figure 4:**
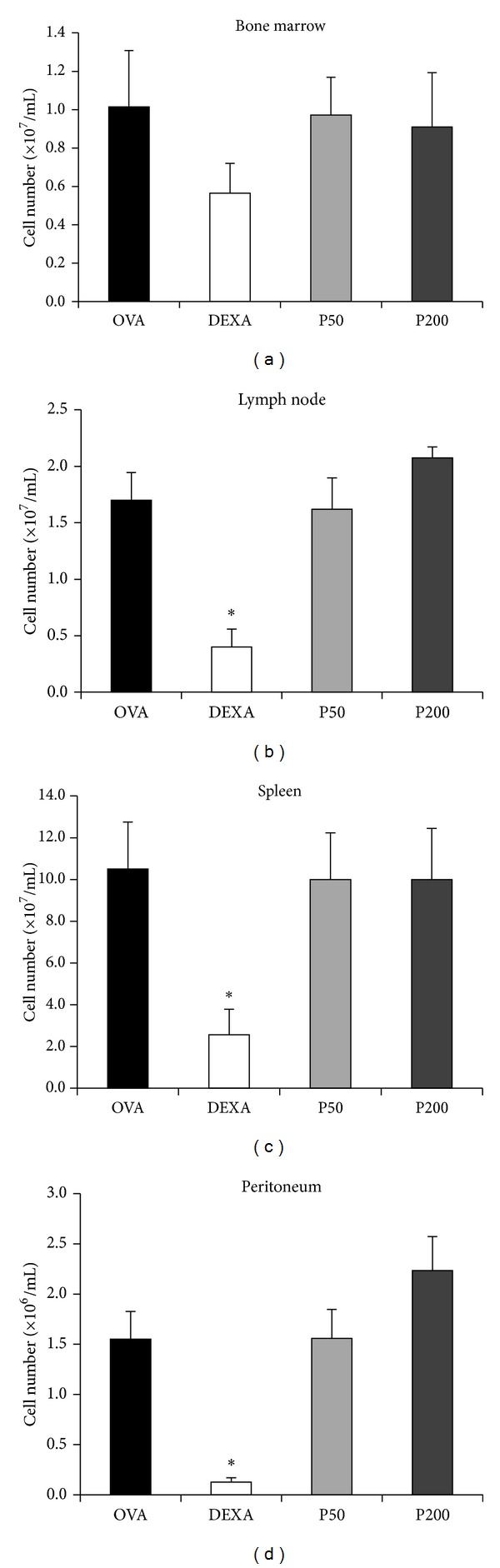
Effect of* S.* aff* postica *PHE treatment on cellular influx to the peritoneal cavity and lymphoid organs of mice immunised and challenged with OVA correspond to bone marrow (a), lymph node (b), and spleen cells (c), respectively. The cells of the peritoneal cavity were harvested and counted to assess cell migration (d). The experiments were performed 24 hours after the last challenge.*P ≤ 0.05 compared to the OVA group.

The PHE treatment did not induce changes in the number of cells in the bone marrow (Figure 4(a)), lymph node (Figure 4(b)), spleen (Figure 4(c)), or peritoneum (Figure 4(d)). Compared to the OVA controls, DEXA treatment significantly decreased the number of cells in the lymph node, spleen, and peritoneum but not in the bone marrow.

## 4. Discussion

The present study shows that the treatment with the PHE given orally reduced significantly the allergic pulmonary inflammation in murine model of asthma. Both treatment with PHE (50 and 200 mg/Kg) reversed the pattern of inflammatory cells in the lung and decreasing the influx of polymorphonuclear inflammatory cells to parenchyma, reversing the pattern of inflammatory cells in the lung and decreasing the influx of polymorphonuclear inflammatory cells to parenchyma. Similarly, it was shown that propolis-treated mice had a reduction in the number of inflammatory cells in the peritoneal bronchoalveolar regions compared with the untreated group [22]. This result is also in accordance with a previous study, which showed that the addition of propolis to the food of asthma patients, as adjunctive therapy in the treatment of this disease, conferred definite advantages by reducing the frequency of crises and the need for rescue medication, possibly improving the patients' immune response [23].

Furthermore, treatment with PHE significantly decreased the IFN-*γ* concentration, which has been considered to be crucial in the pathogenesis of asthma [4, 5]. This result is in accordance with some studies that have demonstrated that the propolis administration over a short term to mice affected both basal and stimulated IFN-*γ* production and the Th1/Th2 balance [24–26], what may be related to its anti-inflammatory properties. Thus, this decrease in IFN-*γ* observed in the PHE-treated groups could induce an improvement in the pulmonary inflammatory condition. This hypothesis is supported by studies that showed that propolis and its products induce inhibition in the synthesis of prostaglandins, leukotrienes, and histamines released* in vitro* by pig lung cells [23] and during induced acute peritoneal inflammation* in vivo* [27].

Besides the inhibitory effect of propolis on IFN-*γ* and on inflammatory cell recruitment, another explanation for the action of propolis in the model used here is its antioxidant effect. Asthma's inflammatory process may be associated with a large release of free radicals because multiple inflammatory cells, including eosinophils, neutrophils, and macrophages, are capable of generating reactive oxygen species at inflammation sites. Consequently, the treatment of asthma with antioxidants has been a successful therapeutic strategy. Lee et al. [28], for example, demonstrated that oxidative stress is a crucial determinant of asthma and that treatment with an antioxidant may be a useful therapeutic strategy. Recent studies have shown that the ethanol extract of propolis is able to interfere with levels of reactive oxygen species [29]. The propolis also reduced the free radical-induced lipid peroxidation as well as increased the activity of superoxide dismutase [30]. It is believed that the free radical- and superoxide-neutralising components of propolis are responsible for the major regenerative and anti-inflammatory effects of this substance [31].

The propolis samples used in the present study showed mainly phenolic acid and total phenol as previously reported [20]. These substances, which have been found in other propolis samples, have been identified as the major compounds with anti-inflammatory activity [32] and can be also related to the beneficial use of propolis in allergies and asthma [33]. Therefore, as the composition of propolis may vary according to the area, it is believed that the antiallergic and anti-inflammatory activities of propolis may depend on a complex interaction among different natural phenolic compounds rather than a single compound [34].

It is important to emphasise that the inhibition of pulmonary inflammation was similar to that observed for treatment with dexamethasone, a potent inhibitor of airway inflammation [6]. However, the immunosuppressive effect of dexamethasone was not observed in the animals treated with propolis, since the number of cells in lymph node, bone marrow, spleen, and peritoneal cavity was not affected in both groups treated with propolis. Thus, the propolis seems to be a more safe treatment when compared to dexamethasone.

Finally, the present study shows that propolis has inhibitory effects on airway inflammation in a murine asthma model, which justifies its use as an alternative/complementary and low cost treatment with virtually no side effects. It is hoped, therefore, that the present work will help to stimulate and enrich discussions and research on* Scaptotrigona* aff.* postica* propolis and will also result in its validation and biological application and advance the preservation of a native species that has shown increased scientific and economic value in recent years.

## 5. Conclusion

Oral treatment with propolis produced by* Scaptotrigona* aff.* postica* reduces the pathology associated with murine asthma, inhibiting both the influx of inflammatory cells to the alveolar space and the systemic progression of allergic inflammation.
